# Treatment-Related Adverse Events of Chimeric Antigen Receptor T-Cell (CAR T) in Clinical Trials: A Systematic Review and Meta-Analysis

**DOI:** 10.3390/cancers13153912

**Published:** 2021-08-03

**Authors:** Wen Lei, Mixue Xie, Qi Jiang, Nengwen Xu, Ping Li, Aibin Liang, Ken H. Young, Wenbin Qian

**Affiliations:** 1 Department of Hematology, The Second Affiliated Hospital, College of Medicine, Zhejiang University, Hangzhou 310009, China; leiwen2017@zju.edu.cn (W.L.); martinxnw@163.com (N.X.); 2 Department of Haematology, The First Affiliated Hospital, College of Medicine, Zhejiang University, Hangzhou 310003, China; 1515013@zju.edu.cn; 3 Department of Medical Oncology, The First Affiliated Hospital, College of Medicine, Zhejiang University, Hangzhou 310003, China; 1515014@zju.edu.cn; 4 Department of Hematology, Tongji Hospital of Tongji University, Shanghai 200065, China; lilyforever76@hotmail.com (P.L.); lab7182@tongji.edu.cn (A.L.); 5 Division of Hematopathology and Department of Pathology, Duke University Medical Center and Cancer Institute, Duke University School of Medicine, Durham, NC 27710, USA; ken.young@duke.edu; 6 Institute of Hematology, Zhejiang University, Hangzhou 310003, China; 7 National Clinical Research Center for Hematologic Diseases, The First Affiliated Hospital of Soochow University, Suzhou 215006, China

**Keywords:** CAR-T, treatment-related adverse events, clinical trial, systematic review

## Abstract

**Simple Summary:**

Successful treatment of hematological malignancies with chimeric antigen receptors T (CAR-T) cells has led to much enthusiasm for the wide clinical usage and development of novel CAR-T therapies. However, it also challenges physicians and investigators to recognize and deal with treatment-associated toxicities. We conducted a systematic review and meta-analysis from 84 eligible study and a total of 2592 patients to identify the comprehensive incidences and severity of CRS and neurological symptoms (NS) as well as the potential differences in AEs across a variety of cancer types, CAR-T targets, and other factors, thereby offering a significant implication on its future application and research.

**Abstract:**

Chimeric antigen receptors T (CAR-T) cell therapy of cancer is a rapidly evolving field. It has been shown to be remarkably effective in cases of hematological malignancies, and its approval by the FDA has significantly increased the enthusiasm for wide clinical usage and development of novel CAR-T therapies. However, it has also challenged physicians and investigators to recognize and deal with treatment-associated toxicities. A total of 2592 patients were included from 84 eligible studies that were systematically searched and reviewed from the databases of PubMed, de, the American Society of Hematology and the Cochrane Library. The meta-analysis and subgroup analysis by a Bayesian logistic regression model were used to evaluate the incidences of therapy-related toxicities such as cytokine release syndrome (CRS) and neurological symptoms (NS), and the differences between different targets and cancer types were analyzed. The pooled all-grade CRS rate and grade ≥ 3 CRS rate was 77% and 29%, respectively, with a significantly higher incidence in the hematologic malignancies (all-grade: 81%; grade ≥ 3: 29%) than in solid tumors (all-grade: 37%; grade ≥ 3: 19%). The pooled estimate NS rate from the individual studies were 40% for all-grade and 28% for grade ≥ 3. It was also higher in the hematologic subgroup than in the solid tumors group. The subgroup analysis by cancer type showed that higher incidences of grade ≥ 3 CRS were observed in anti-CD19 CAR-T therapy for ALL and NHL, anti-BCMA CAR-T for MM, and anti-CEA CAR-T for solid tumors, which were between 24–36%, while higher incidences of grade ≥ 3 NS were mainly observed in CD19-ALL/NHL (23–37%) and BCMA-MM (12%). Importantly, subgroup analysis on anti-CD19 CAR-T studies showed that young patients (vs. adult patients), allologous T cell origin (vs. autologous origin), gamma retrovirus vector, and higher doses of CAR-T cells were associated with high-grade CRS. On the other hand, the patients with NHL (vs ALL), administered with higher dose of CAR-T, and adult patients (vs. young patients) had an increased incidence of grade ≥ 3 NS events. This study offers a comprehensive summary of treatment-related toxicity and will guide future clinical trials and therapeutic designs investigating CAR T cell therapy.

## 1. Introduction

Chimeric antigen receptor T (CAR-T) cell therapy employs autologous or allogeneic genetically engineered T cells for combatting cancer. It has demonstrated unexpected success in treatment-refractory patients. CAR-engineered T cells targeting CD19 has shown remarkable efficacy in patients with recurrent/refractory CD19^+^ B-cell malignancies in recent years [1,2,3,4,5]. Phase I and II trials that study the effect of CAR-T on B-cell maturation antigen (BCMA) or CD22 have shown potent antitumor activity for multiple myeloma (MM) and acute lymphoblastic leukemia (ALL), respectively [6,7,8,9,10,11,12]. Although the efficacy of CAR-T in solid tumors has been limited, an unprecedented number of CAR-T cells trials on solid tumors have been finished or are ongoing [13,14,15,16,17].

CAR-T cell therapy has a unique set of toxicities related to the activation of the immune system. Thus, it is distinct from chemotherapies, targeted small molecule drugs, and other immunotherapies. Cytokine-release syndrome (CRS), CAR-T-cell-related encephalopathy syndrome (CRES), and other adverse events (AEs) occurring after CAR-T cell treatment should be considered in clinical practice. Although safety levels of CAR-T therapy are generally acceptable, the variability in incidences and severity of AEs in different clinical trials employing commercially and locally produced CAR-T cells may be attributed to the difference in the construct, dosage, intensive lymphodepletion, tumor burden, and clinical practices at various centers [2,18,19]. Due to the specific toxicity profiles and the life-threatening potential of CAR-T therapy found on clinical trials, a systematic review is needed to evaluate its variable safety profiles. In this study, we performed a systematic review and meta-analysis of published clinical trials on the treatment-related AEs that occurred early after CAR T-cell infusion. Using the rate meta-merge and the novel Bayesian approach, we investigated the comprehensive incidences and severity of CRS and neurological symptoms (NS). Further, we quantified potential differences in AEs across a variety of cancer types, CAR-T targets, and other factors, thereby offering a significant implication on its future application and research.

## 2. Materials and Methods

This systematic review was conducted with adherence to the Preferred Reporting Items for Systematic Reviews and Meta-Analyses (PRISMA) guidance [20].

### 2.1. Data Sources

A systematic search of the literature was conducted to identify published clinical trials on the treatment-related AEs of CAR-T therapy. The PubMed, the EMBASE, and the American Society of Hematology and Cochrane Library databases were systematically searched for eligible studies. The search time was from database inception to October 1, 2020. A combination of free-text words and MeSH terms was used as follows: (Chimeric Antigen Receptor/CAR-T/engineered T cell/modified T cell) AND (adoptive/therapy/treat/immunity/immunotherapy). Reference lists from eligible studies were thoroughly searched for potentially relevant studies.

### 2.2. Study Selection, Meta-Analysis Inclusion Criteria, and Data Extraction

Identified publications were carefully screened. Two reviewers (Qi Jiang and Mixue Xie) screened all identified publications based on our inclusion criteria. In the event of disagreement between the two reviewers, we obtained and inspected the full-text article independently. The references of relevant published trials, case reports, and meeting abstract were included for meta-analysis. In total, 84 studies were included in the final analysis. The inclusion criteria were as follows: (1) cancer therapy clinical trials or researches that involve more than three patients; (2) trials or researches that focus on CAR-T (NK) therapy; (3) studies reporting data on treatment-related AEs; and (4) studies published in English. Extracted data included the following: (1) study characteristics (author, publication time, research region, and study type); (2) patient characteristics (number, age, gender, and disease); (3) CAR-T type and manufacturing process (T-cell original, vector, dose, and co-stimulation domain); and (4) outcome measures (number of AEs and criteria for AE reporting in the publication; all-grade AE and grade 3 or higher AE data were both extracted). When there were multiple publications reporting on the same study population, the one with the most updated and/or comprehensive AE data was selected.

### 2.3. Statistical Analyses

Pooled estimates of treatment-related AEs were computed when there was sufficient reporting of these measures. The overall pooled effects assessment was conducted using a fixed-effects model. In the case of significant heterogeneity, a random-effects model was used. Heterogeneity in the results of the trials was assessed using the χ^2^ test of heterogeneity and the I^2^ measure of inconsistency. Heterogeneity was considered present when the *p* value of the Cochran Q test was <0.05 and the I^2^ statistic was >50%. The rate meta-merger method was also applied to the subgroup analysis, including the cancer type and the CAR-T type, by study-level moderators.

The Bayesian logistic regression model was applied for subsequent subgroup analysis of anti-CD-19 CAR-T studies based on the type of cancer (ALL and Non-Hodgkin Lymphoma [NHL]), age group (child, young adult, and adult), T-cell origin (autologous and allogeneic), and construct type (vector and co-stimulatory domain types) to investigate the potential underlying relationships that may explain some of the variability in AE rates. We categorized variables such as cancer type and construct type among others according to sub-group definitions and transformed the dependent variable (incidence of CRS and NS) into a binary variable (occur = 1, not occur = 0). All of the included research data, including the Bayesian logistic regression model with a non-informative prior, became a patient-level dataset. With a logit transformation (logit(z) = log(z) − log(1 − z)) on the incidence probability, we assumed normal distributions for the patient-level effects. A non-informative prior distribution was proposed for the mean parameters of normal distributions. Based on sufficient information in the dataset, we set the standard deviation of the priors to 10 for all variables. The estimate of the parameters of the posterior distribution was used by the Markovchain Monte Carlo algorithm. Before the operation, the burning of the annealing number was set to 5000 times. The number of iterations of McMcsize was set to 50,000. Due to the large number of iteration sets, the adaptation was also set for the number of updates and simulations. Using the Bayesian logistic regression, the odds ratios (ORs) value of the comparison between the subgroup parameters, one of which was the control parameter, was obtained. When the OR value was >1, it indicated that the test parameters were correlated with the high incidence of AE and vice versa. All statistical analyses were performed using the meta-analysis command in the STATA (version 14.0 for Windows; Stata Corp LP, College Station, TX, USA).

## 3. Results

### 3.1. Eligible Studies and Characteristics

**Figure 1:**
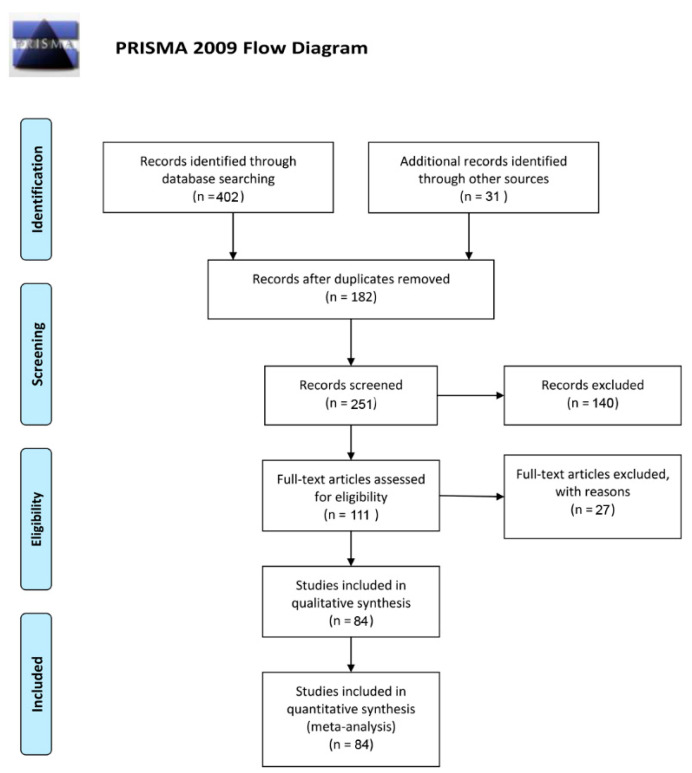
Flow diagram of the study selection process.

The procedure for the systematic literature review is shown in the PRISMA flow diagram (Figure 1). After screening and eligibility assessment, our study included 84 clinical trials with a total of 2592patients (Table 1). There were 19 kinds of CAR-T cells targeting different tumor-cell antigens. The kinds of CAR-T cells included the following: CD19, CD20, CD22, CD30, CD33, LeY Antigen, BCMA, and natural killer group 2D (NKG2D) for hematological malignancies, and carcinoembryonic antigen (CEA), epidermal growth factor receptor (EGFR), GD2, IL13 receptor α2, epidermal growth factor receptor 2 (HER2), mesothelin, prostate specific membrane antigen (PSMA), and tumor-associated glycoprotein-72 (TAG72) for solid tumors.

The identified clinical trials involved treatment of seven categories of hematologic malignancies, including ALL (*n* = 24), B-cell NHL (*n* = 21), MM (*n* = 7), Hodgkin lymphoma (HL) (*n* = 3), acute myeloid leukemia (AML) (*n* = 1), chronic lymphocytic leukemia (CLL) (*n* = 1), and mixed cancer types (*n* = 14). The identified clinical trials also involved treatment of 11 types of solid tumors, including colorectal cancer (CRC) (*n* = 2), liver cancer, NSCLC (*n* = 1), biliary tract cancer (*n* = 1), glioblastoma (*n* = 3), neuroblastoma (*n* = 1), sarcoma (*n* = 1), pancreatic cancer (*n* = 1), prostate cancer (*n* = 1), and mixed cancer types (*n* = 2). Most of these studies were performed in either the United States (46 studies, 52.8%) or China (17 studies, 29.5%).

### 3.2. Overall Incidence of CRS and Non-Hematological AEs

The lympho-depletion protocol was generally required in most CAR-T therapies leading to subsequent hematological toxicity. Therefore, the data on treatment regarding the hematological toxicity reported in these trials were not analyzed in this review. Consequently, we analyzed the incidences of NS and non-hematological AEs that were likely CAR-T cell therapy-related. Among the 84 included literatures, CRS classification criteria were varied: 45 studies adopted the 2014 Lee criteria, 1 study adopted the American Society for Transplantation and Cellular Therapy (ASTCT) criteria, also known as 2019 Lee criteria, 15 studies adopted the Common Terminology Criteria for Adverse Events version (CTCAE) criteria, 8 studies adopted the Penn criteria, and 2 studies adopted the Memorial Sloan Kettering Cancer Center (MSKCC) criteria. The remaining 17 literatures did not clarify the CRS grading criteria. A total of 1244 of 1700 patients from 55 studies developed CRS of any grade, with a pooled prevalence of 77% (95% CI: 74–80%), while 567 of 2239 patients reported from 58 studies suffered a CRS grade of 3 or higher, with a pooled prevalence of 29% (95% CI: 24–34%). We observed that all grade CRS toxicities occurred as frequently as 81% (95% CI: 78–85%) in patients with hematologic malignancies, whereas it occurred as frequently as 37% (95% CI: 0–91%) in solid tumor patients. Similarly, the CRS rate (grade ≥ 3) was higher for blood cancer patients (29%, 95% CI: 24–34%) as compared with patients with solid tumors (19%, 95% CI: 8–31%).

**Figure 2:**
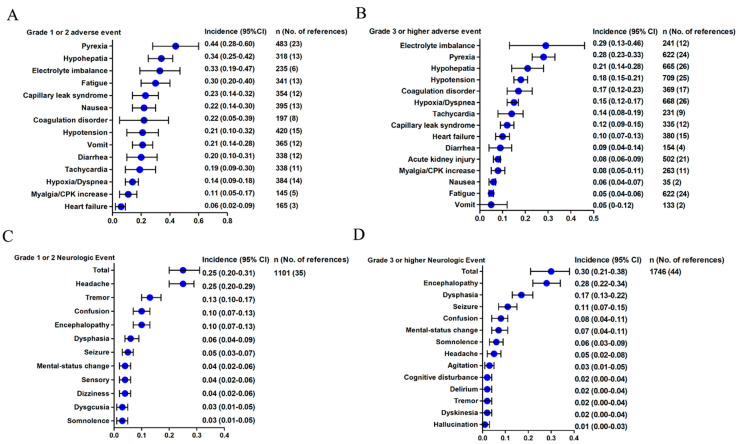
Overall incidence of non–hematological AEs and NS–related AEs. (**A**). Grade 1 or 2 adverse event; (**B**). Grade 3 or higher adverse event; (**C**). Grade 1 or 2 Neurologic Events; (**D**). Grade 3 or higher Neurologic Event.

For the non-hematological AEs, the most common grade 1–2 AEs were pyrexia (44%; 95% CI, 28–60%), hypohepatia (34%; 95% CI, 25–42%), electrolyte imbalance (33%; 95% CI, 19–47%), fatigue (30%; 95% CI, 20–40%), and capillary leak syndrome (23%; 95% CI, 14–32%) (Figure 2A). The most common ≥ 3 grade AEs were electrolyte imbalance (29%; 95% CI, 13–46%), pyrexia (28%; 95% CI, 23–33%), hypohepatia (21%; 95% CI, 14–28%), hypotension (18%; 95% CI, 15–21%), and coagulation disorder (17%; 95% CI, 12–23%) (Figure 2B).

### 3.3. Overall Incidence of NS-Related AEs

A total of 544 of 1456 patients from 39 studies and 611 of 2044 patients from 45 studies developed at least one NS of any grade and grade 3 and above, respectively. The pooled estimate NS rate and 95% CI from the individual studies were 40% (95% CI, 26–53%) for any grade and 28% (95% CI, 21–35%) for grade 3 and above. The subgroup-analysis result confirmed that the any NS rate was higher for patients with hematologic malignancies (any grade 40%, 95% CI, 25–54%) as compared with patients with solid tumors (any grade 32%, 95% CI, 24–41%).

There were 35 trials, including 1101 patients, that had grade 1–2 NS of AEs. As shown in Figure 2C, the overall estimate of grade 1–2 NS rate and the 95% CI were 25% (95% CI, 20–31%). The most frequent grade 1–2 NS were headache (25%; 95% CI, 20–29%), tremor (13%; 95% CI, 10–17%), confusion (10%; 95% CI, 7–13%), encephalopathy (10%; 95% CI, 7–13%), dysphasia (6%; 95% CI, 4–9%), and seizure (5%; 95% CI, 3–7%) (Figure 2C). A total of 44 studies with 1,746 patients reported grade 3 or higher NS of AEs. The overall estimate of NS rate and 95% CI from the individual studies was 30% (95% CI, 21–38%). The most common grade 3 or higher AEs were encephalopathy (28%; 95% CI, 22–34%), dysphasia (17%; 95% CI, 13–22%), seizure (11%; 95% CI, 7–15%), confusion (8%; 95% CI, 4–11%), mental-status change (7%; 95% CI, 4–11%), somnolence (6%; 95% CI, 3–9%), and headache (5%; 95% CI, 2–8%) (Figure 2D).

### 3.4. Incidence of Treatment-Related Deaths

**Figure A1:**
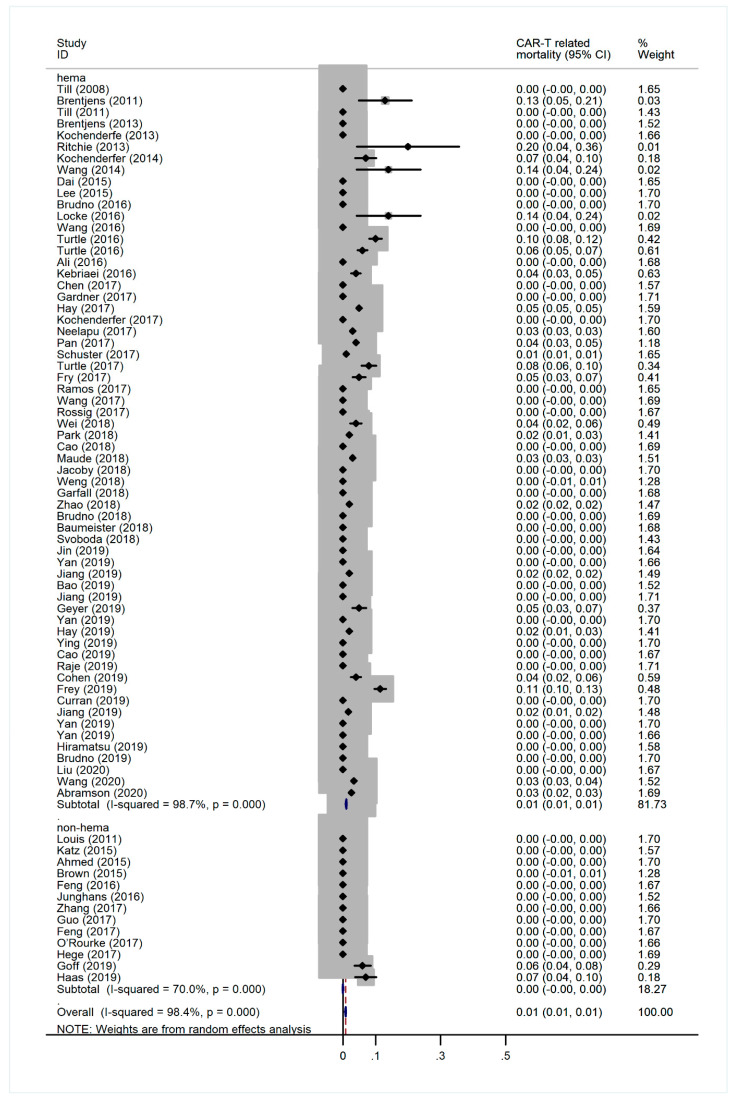
Overall incidence of CAR-T–related mortality.

Among the 84 studies, 62 hematological studies and 13 non-hematological studies reported whether any CAR-T-related deaths occurred. Among these, 29 studies reported at least one treatment-related death, with a total of 53 such deaths reported. The overall pooled incidence of treatment-related deaths was 1%, which was higher in patients with hematologic malignancies (1%, 95% CI: 1–1%) than that of patients with solid tumors (0%, 95% CI: 0–0%) (Figure A1).

As shown in Table A1, the most common cause of treatment-related death (*n* = 53) was CRS (23, 43.4%. Other common causes were neurological symptoms (8, 15.1%), sepsis (5, 9.4%), and hemorrhage (8, 15.1%). Respiratory causes (5, 9.4%), cardiovascular (3, 5.6%), and aGVHD (1, 1.9%) were the other common causes.

### 3.5. Subgroup Analysis of AE Incidence by Cancer Type

**Figure 3:**
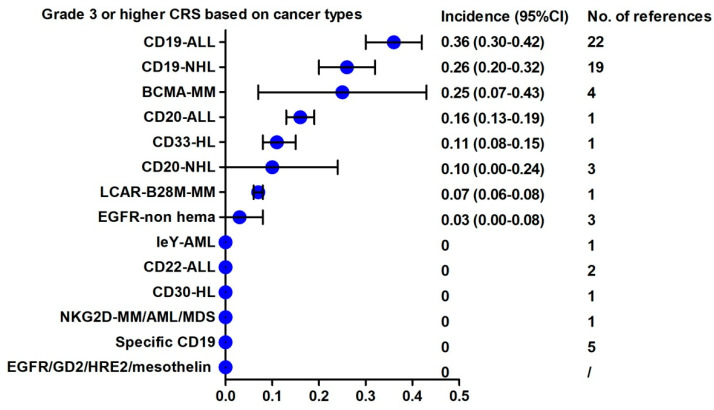
Rank of the incidence of grade 3 or higher CRS based on cancer types.

**Figure A2:**
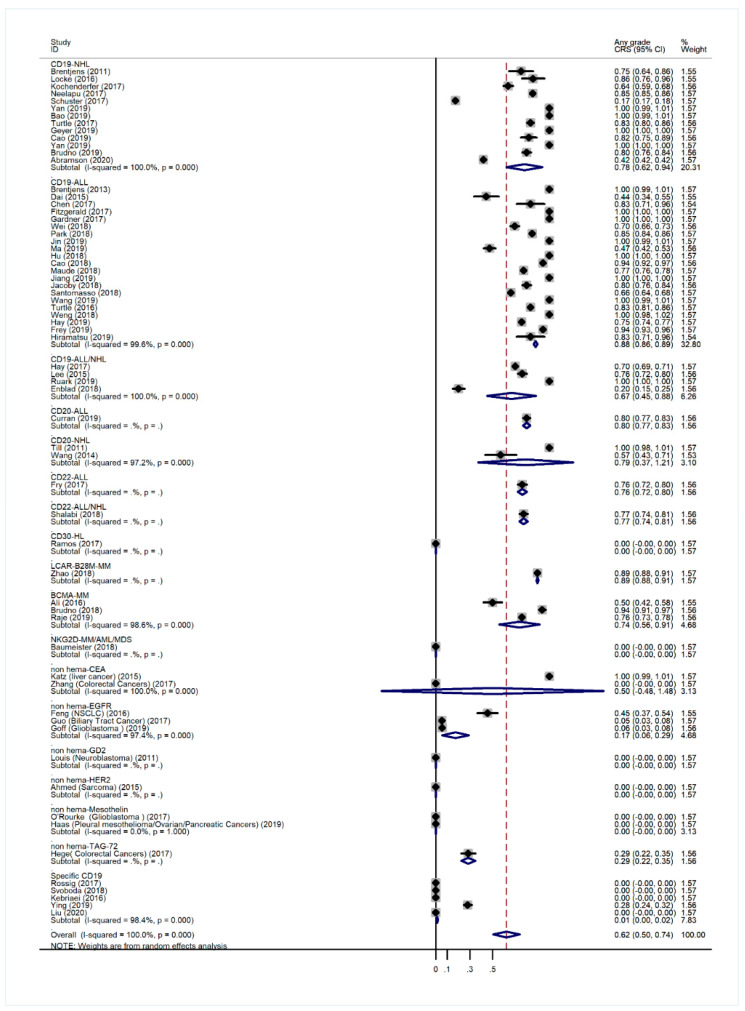
Subgroup analysis of any grade CRS event incidence by cancer type.

**Figure A3:**
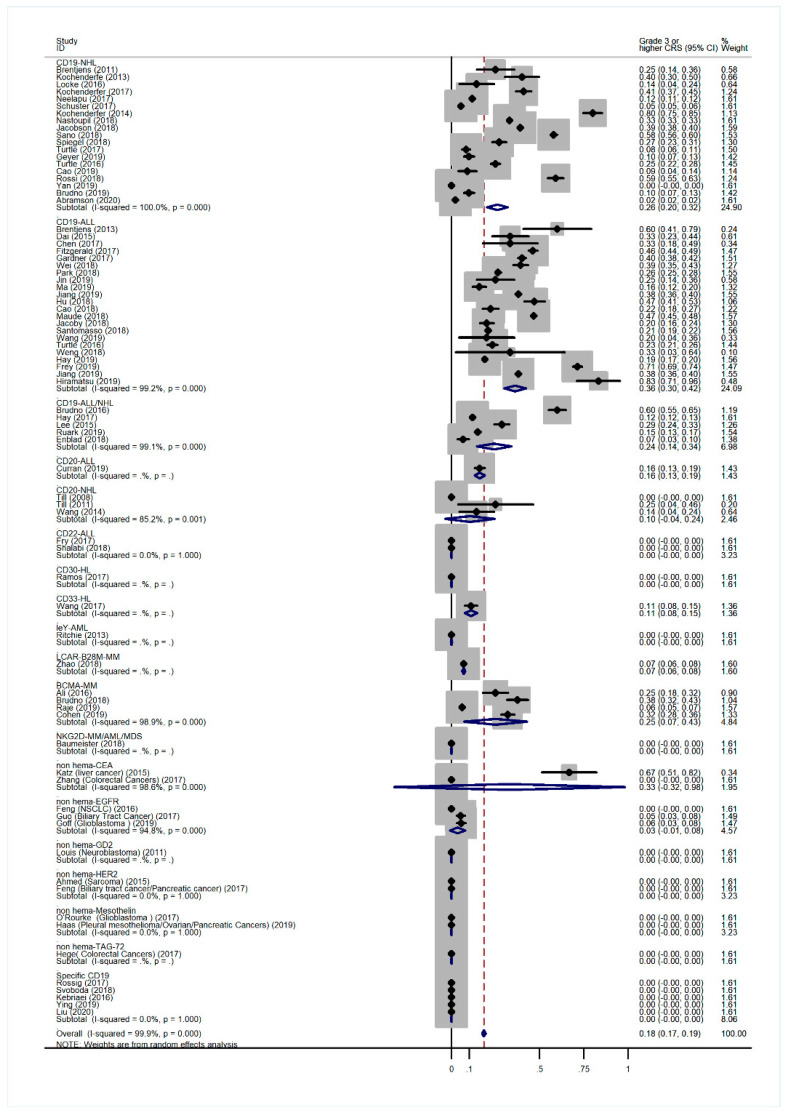
Subgroup analysis of ≥3 grade CRS event incidence by cancer type.

Based on cancer type and CAR-T type, we classified the 84 identified studies into 18 different categories: 12 for hematological malignancies including CD19-NHL, CD19-ALL, CD19-ALL/NHL, CD20-NHL, CD22-ALL, CD30-HL, CD33-HL, leY-AML, LCAR-B28-MM, BCMA-MM, NKG2D-MM/AML/MDS, and specific CD19; and 6 for solid tumors including CEA, EGFR, GD2, HER2, methesolin, and TAG-72. As shown in Figure A2, higher any grade CRS event incidences were observed in CD19-ALL (88%; 95% CI, 86–89%), CD19-NHL (78%; 95% CI, 62–94%), CD20-NHL (79%; 95% CI, 37–121%), CD20-ALL (80%; 95% CI, 77–83%), CD22-ALL (76%; 95% CI, 72–80%), BCMA-MM (74%; 95% CI, 56–91%), and CEA (50%; 95% CI, 48–148%). Relatively lower CRS event rates of any grade (less than 20%) were observed in CD30-HL, NKG2D-MM/AML/MDS, EGFR, GD2, HER2, and mesothelin. As shown in Figure 3, higher mean ≥ 3 grade CRS event incidences were observed in the anti-CD19 CAR-T treatment of ALL and NHL, and anti-BCMA CAR-T of MM, which ranged between 25% and 36%. There were rarely no CRS events of grade 3 or higher observed in CD22-ALL, CD30-HL, leY-AML, NKG2D-MM/AML/MDS, and in solid tumor-related CAR-T treatment, including targets of EGFR, GD2, HER2, mesothelin, and TAG-72. These data suggest that the mean incidences of all-grade and grade 3 or worse CRS events were higher in hematologic malignancies compared to solid tumors. In hematologic malignancies, the highest incidence and the most severe CRS were observed in the anti-CD19 CAR-T treatment of ALL and NHL. Recently, a number of studies have made a methodological breakthrough in the study of alternative anti-CD19 CAR-T therapy to reduce serious AEs. The methodological breakthroughs included origin selecting from EBV-specific CTL cells [61], vector selecting non-viral RNA [62], CAR-T combing with SB transposon/transposase system [63], and CAR-T targeting CD19-BBz (84) or CAR-NK targeting CD19 [69]. As shown in Figure A2 and Figure A3, the incidence of CRS in either ≥ grade 3 or any grade was significantly decreased in alternative CD19-CAR-T treatment compared with the traditional second generation CD19 CAR-T therapy.

**Figure 4:**
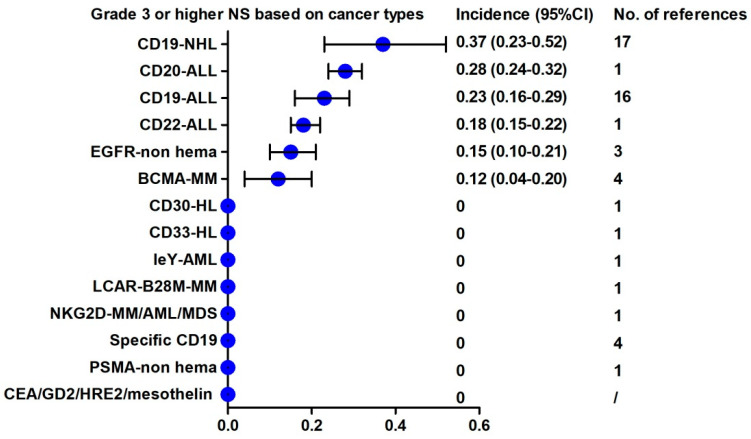
Rank of the incidence of grade 3 or higher NS based on cancer types.

**Figure A4:**
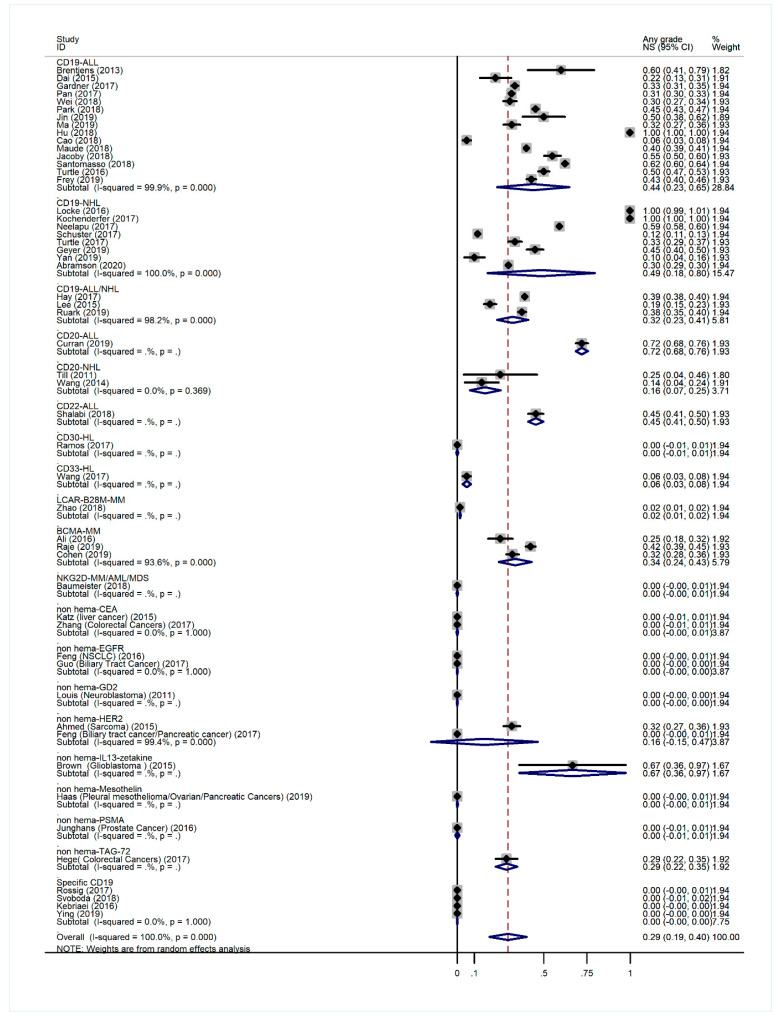
Subgroup analysis of any grade NS event incidence by cancer type.

**Figure A5:**
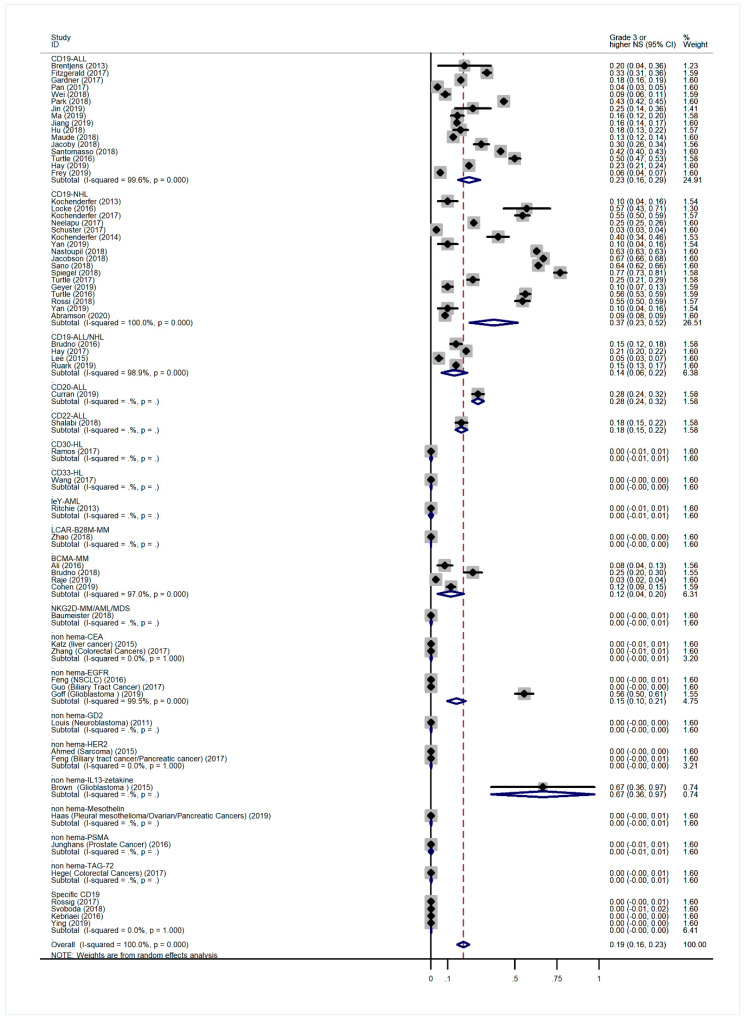
Subgroup analysis of ≥3 grade NS event incidence by cancer type.

NS of any grade and grade 3 or higher also conducted a subgroup analysis by cancer type. As shown in Figure A4, higher any grade NS event incidences were observed in CD19-ALL (44%; 95% CI, 23–65%), CD19-NHL (49%; 95% CI, 18–80%), CD20-ALL (72%; 95% CI, 68–76%), CD22-ALL (45%; 95% CI, 41–50%) and BCMA-MM (34%; 95% CI, 24–43%). Higher grade 3 or higher NS event incidences were also observed in CD19-NHL (37%; 95% CI, 23–52%), CD20-ALL (28%; 95% CI, 24–32%), CD19-ALL (23%; 95% CI, 16–29%), CD22-ALL (18%; 95% CI, 15–22%), BCMA-MM (12%; 95% CI, 4–20%) and anti EGFR CAR T of solid tumors (15%; 95% CI, 10–21%) (Figure 4 and Figure A5). There were no NS events reported in solid tumors.

### 3.6. Subgroup Analysis of Anti-CD19 CAR-T-Related AE Incidence

Due to the great heterogeneity of CAR-T-related AEs in different tumors and therapeutic targets, we conducted further subgroup analysis on anti-CD19 CAR-T studies with the most sufficient data (*n* = 51). The subgroup analysis factors included cancer types (ALL vs. NHL), region (China vs. USA), age (adult ≥ 20 years old vs. child and young ≤ 30), T-cell origin (autologous vs. allologous), co-stimulation domain (4-1BB vs. CD28 vs. 4-1BB combined with CD28), vector (gammaretrovirus vs. lentivirus vs. retrovirus), lympho-depletion protocol (Cy vs. Cy + Flu), and dose (10^5^–10^6^/kg vs. 10^6^/kg vs. 10^6^–10^8^/kg).

**Figure 5:**
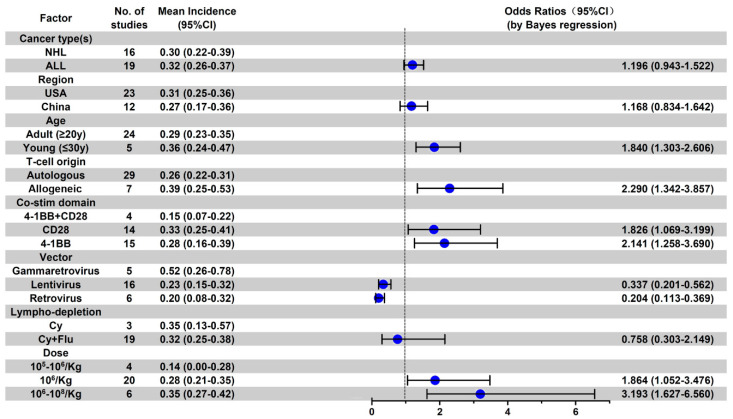
Subgroup analysis of anti-CD19 CAR-T-related ≥ 3 Grade CRS Event Incidence.

**Figure A6:**
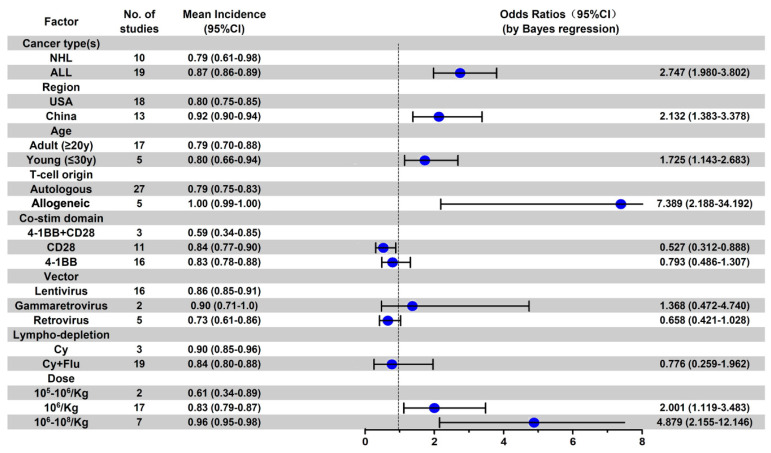
Subgroup analysis of anti-CD19 CAR-T-related any grade CRS event incidence.

The subgroup analysis using the Bayesian logistic regression showed that the higher mean incidences of all-grade CRS events were observed in the following subgroups: ALL patients (OR 2.747; 95% CI, 1.980–3.802), patients in the China region (OR 2.132; 95% CI, 1.383–3.378), young patients (OR 1.725; 95% CI, 1.143–2.683), patients treated with allologous CAR-T cell (OR 7.389; 95% CI, 2.188–34.192), and patients treated with higher CAR-T cell infusion dose (10^6^/kg vs. 10^5^/kg, OR 2.001; 95% CI, 1.119–3.483; 10^5^/kg vs. 10^6–8^/kg, OR 4.879; 95% CI, 2.155–12.146) (Figure A6). When performing subgroup analysis on ≥3 grade CRS, young patients (OR 1.84; 95% CI, 1.303–2.606), allologous T cell sources (OR 2.29; 95% CI, 1.342–3.857), and CAR-T cell infusion doses were associated with higher incidences. In addition, the gammaretrovirus vector increased ≥ 3 grade CRS incidence as compared with the lentivirus or retrovirus. The third-generation CAR-T treatment with CD28 in combination with 4-1BB co-stimulation domains can significantly reduce the incidence of ≥3 grade CRS as compared with CD28 or 4-1BB co-stimulation (Figure 5).

**Figure 6:**
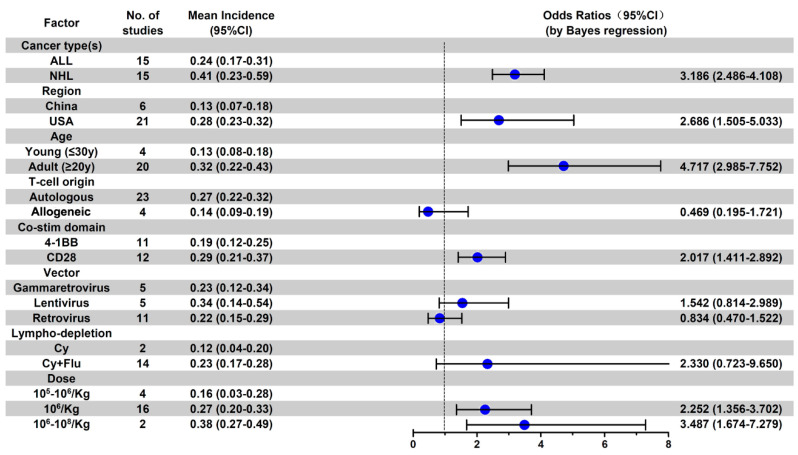
Subgroup analysis of anti-CD19 CAR-T-related ≥ 3 grade NS event incidence.

**Figure A7:**
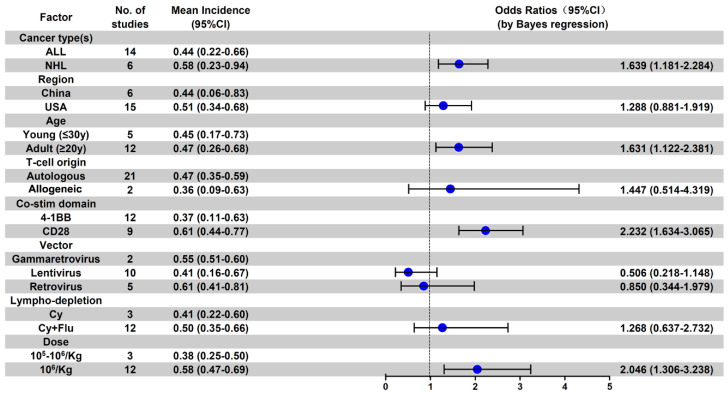
Subgroup analysis of anti-CD19 CAR-T-related any grade NS event incidence.

As shown in Figure A7, higher mean incidences of all-grade NS events were observed in the following subgroups: NHL patients (OR 1.639; 95% CI, 1.181–2.284), adult patients (OR 1.631; 95% CI, 1.122–2.381), patients treated with the CAR-T with CD-28 co-stimulation domain (OR 2.232; 95% CI, 1.634–3.065), and patients treated with higher a CAR-T cell infusion dose (10^6^/kg vs. 10^5^/kg, OR 2.046; 95% CI, 1.306–3.238). For grade 3 or higher NS events, in addition to the above factors, the patients in the United States region had higher ≥ grade 3 NS rate (OR, 2.686; 95% CI, 1.50–5.033) as compared with patients in China (Figure 6).

## 4. Discussion

CAR-T therapy, one of the most promising tumor treatments, has been approved by the Food and Drug Administration (FDA) for the treatment of patients with ALL and large B-cell lymphoma (Tisagenlecleucel and Yescarta) in 2017 [27,30]. This means that CAR-T therapy has been affirmed as an official and clinical treatment option for tumor. The number of CAR-T studies investigating different cancer-specific target has been rapidly increasing. As of January 2021, the information from Clinicaltrails.gov has showed that more than 500 clinical studies on CAR-T therapy have been conducted globally. However, promising results have been threatened by safety considerations. A comprehensive analysis of common CAR-T-related AEs reported in clinical trials is needed. The results of such a comprehensive analysis provide important references for clinicians. Previous meta-analysis focused on certain AEs such as incidence of CRS and NS, but most of them focused on the summary of a single target or disease such as CD19 CAR-T and B-ALL. There was no systematic summary based on different tumor types and targets [86,87]. In addition, previous meta-analysis did not show the incidence of common adverse reactions of CRS and NS such as coagulation dysfunction and encephalopathy of CRS and NS. There was also lack of large sample studies to explore the factors that potentially influence CAR-T adverse reactions. We performed this systematic review, which aimed to summarize CAR-T associated AEs in patients with hematological malignancies and solid tumors from published clinical trials. To our knowledge, this is the largest and most comprehensive meta-analysis of treatment-related AEs observed and encountered in CAR-T therapy. In our meta-analysis, we used common CRS and NS as the endpoints for data collection and combined traditional meta-analysis methods and Bayesian statistical methods for analysis. Based on study-level data, the meta-merging method was used to summarize the incidence of CAR-T AEs and for subsequent subgroup analysis by different tumor types and CAR-T targets. For the exploration of potential related factors in AEs, we used the Bayesian logistic regression model based on the individual-level data to provide a more realistic statistical estimate.

From the perspective of patient consultation, there were several findings from our study that deserve attention. As the most common event after CAR-T cell immunotherapy, CRS occurred in over 70% of patients. CRS occurred in 80% of hematological malignancies and in 40% of solid tumors. In more than half of the patients, it was mild to moderate and resolved within a few days. Eventually, nearly 30% of patients developed severe CRS (more than grade 3) and received tocilizumab or dexamethasone. Although pyrexia was the most common non-hematological manifestation, serious AEs included electrolyte imbalances, hypohepatia, hypotension, coagulation dysfunction, and hypoxia, which required more attention from clinicians. For grade 3 or more electrolyte imbalance, as many as 207 cases provided the specific electrolyte data, of which 50.2% were hypophosphatemia, followed by hyponatremia and hypokalemia, each accounting for 19.3% and 18.8%. Of the 82 cases providing specific manifestations of liver damage, more than half of the patients had an increase in aspartate aminotransferase. On the other hand, those with elevated alanine aminotransferase and bilirubin accounted for only 26.8% and 17.1%, respectively. The other most common specific manifestations associated with CRS were coagulation dysfunction, hypotension, hypoxia, and capillary leakage syndrome, all with incidences of more than 10%. It was worth noting that coagulation dysfunction, as a common manifestation of CRS, might develop into DIC in severe cases and was one of the important causes of CAR-T-related deaths, accounting for approximately 10% in our report. Of the 369 patients from 17 trails, we reported 22% of patients with grade 1 to 2 coagulation dysfunction and 17% of patients with grade 3 or more coagulation dysfunction. Prolonged APTT and hypofibrinemia were main clinical manifestations. Our data were consistent with those reported by Jiang et al. [88]. Their data showed that 56% (30/53) of patients with r/r B-cell acute lymphoblastic leukemia (B-ALL) developed coagulopathy after receiving split infusions of anti-CD19 CAR-T cells. Half of them should have been diagnosed with DIC. A total of 14 patients successfully recovered from DIC through replacement and anticoagulation treatment. The study also clarified the changes in plasma concentrations of tissue factor (TF) and platelet endothelial cell adhesion molecule-1 (PECAM-1), and suggested that these have important significance in the etiology and pathogenesis of CAR-T related coagulopathy.

NS, another common AE of CAR-T therapy, is also worth disclosing to patients. Approximately one-third of patients treated with CAR-T in clinical trials developed at least one NS of any grade. In hematologic malignancies, the incidence (40%) was slightly higher than in solid tumors (32%). According to our meta-analysis, the most common mild to moderate NS symptoms were headache, tremor, and confusion. The most common severe NS symptoms were encephalopathy, dysphasia, and seizure. Rubin et al. characterized NS associated with CAR-T therapy in a consecutive series of 100 patients up to t months post transfusion [89], and found that focal neurological deficits were frequently observed after CAR-T therapy and were associated with regional EEG abnormalities, fluorodeoxyglucose-positron emission tomography hypometabolism, and elevated velocities on transcranial Doppler ultrasound. In contrast, structural imaging was typically normal. Although CAR-T therapy resulted in common CRS and NS, which were life-threatening in severe cases, the mortality rate associated with CAR-T therapy in our study was relatively low (not exceeding 1%) due to early pharmacological intervention.

Although previous studies in the literature have reported the efficacy, including the overall response rate and the complete response rate, of CAR-T therapy for different tumors with different targets, there are few comprehensive analyses for the incidences of CRS and NS based on different tumors and targets of the therapy. A previous meta-analysis suggested that the CRS rate was significantly higher in patients with hematologic malignancies than that in patients with solid tumors (any grade 67% vs. 35%) [86]. Furthermore, the NS rate was slightly higher in patients with hematologic malignancies than that in patients with solid malignancies (any grade 9% vs. 6%). The incidence of any grade CRS in our study was 81% in hematological malignancies and 37% in solid tumors, which was consistent with the result of the above study [86]. However, the incidences of any grade NS in our study (40% in hematologic and 32% in solid tumor) were significantly higher than in previous meta-analysis. The reason may be due to the addition of studies published in 2019. NS, as common AEs of CAR-T, have gradually been valued and recorded by researchers since then. The reason why CRS rate of hematologic malignancies was significantly higher than that of solid tumors has not yet been clearly elucidated. It has been previously reported the ORR was significantly higher in patients with hematologic malignancies (about 71%) than in patients with solid tumors (about 20%) [86]. We believed that the incidence of CRS was likely to be related to the efficacy of CAR-T therapy. The mechanism of CRS generation was derived from the mechanism of CAR-T treatment. Since it was difficult to unify the efficacy criterions for different solid tumors and hematological malignancies, our study did not analyze the correlation between CRS and efficacy. However, Grigor et al. conducted a post-hoc analysis using the scatter plot to visually investigate a possible relationship between CRS and complete response in B-cell malignancies, which demonstrated no relationship [87]. Similar to the efficacy, the evaluation criteria for tumor burden were difficult to unify in different hematological and solid tumors. However, in B-cell malignancies, studies have confirmed that the high incidence of CRS was associated with higher tumor burden [18,19]. We then focus on different CAR-T targets. It can be seen that the higher incidences of CRS and NS were observed in patients receiving anti-CD19 CAR-T therapy compared to patients treated with other targets of CAR-T therapies. It was also worth noting that patients with B-ALL had a higher incidence of CRS than patients with B-lymphoma, while patients with B-lymphoma had a higher incidence of NS, followed by MM patients treated with BCMA-targeted CAR-T therapy. In most patients with solid tumors, grade 3 or higher CRS and NS were rare. The CEA target study [78] (*n* = 6), EGFR target study [81] (*n* = 18), and IL13 target study [13] (*n* = 3) failed to draw valid conclusions due to the small number of included cases.

The Bayesian logistic regression models were applied for further subgroup analysis to investigate the potential factor affecting CAR-T related AEs. Due to the great heterogeneity among different tumors and therapeutic targets, we conducted the subgroup analysis only on anti-CD19 CAR-T studies. The subgroup analysis factors included patients’ characteristics such as cancer types (ALL vs. NHL), region (China vs. USA), age (adult ≥ 20 years old vs. child and young ≤ 30 years old), and factors related to the process of CAR-T production such as T-cell origin (autologous vs. allologous), co-stimulation domain (4-1BB vs. CD28 vs. 4-1BB combined with CD28), vector (gammaretrovirus vs. lentivirus vs. retrovirus), lympho-depletion protocol (Cy vs. Cy+Flu), and dose (10^5^–10^6^/kg vs. 10^6^/kg vs. 10^6^–10^8^/kg). Hay et al. [18] identified biomarkers of severe CRS in 133 adult patients who received CD19 CAR-T cells and found high tumor burden in bone marrow, lymphodepletion with cyclophosphamide and fludarabine, higher CAR-T cell dose, thrombocytopenia before lymphodepletion, and CAR-T cell manufacturing without selection of CD8^+^ central memory T-cells as independent predictors of CRS. Consistent with the above results, we found that a higher CAR-T cell infusion dose was an important factor increasing the incidences of CRS and NS. Contrary to the above results, the lymphodepletion regimen, which either included fludarabine or not, did not affect the occurrence of CRS or NS. The Bayesian logistic analysis also found that CRS had a higher incidence in B-ALL patients than in B-lymphoma patients, in which any grade was statistically significant. NS had a higher incidence in B-lymphoma patients than in B-ALL patients, in which any grade and grade 3 or higher were statistically significant. In addition, in our meta-analysis, child and young patients were at risk for CRS while adults had a higher risk for NS. The ethnic or geographic differences affected CAR-T related toxicity. A higher NS incidence and a lower CRS rate were observed more in the USA than in China. In terms of factors related to the production process of CAR-T cells, we found that allologous T-cell origin was an important factor in the occurrence of CRS. Moreover, the third generation of CAR-T by using CD28 combined with 4-1BB co-stimulations significantly decreased CRS rate as compared to CD28 or 4-1BB co-stimulation. The findings of our subgroup analyses, especially on factors such as age, region, and co-stimulations, need to be validated prospectively in large scale studies. Additional understanding of the mechanisms that resulted to severe CRS would have facilitated testing of interventions to prevent or reverse toxicity and improve the safety of CAR-T cells.

It’s worth noting that 84 clinical trials analyzed in our study had used different grading scales to evaluate CRS and NS, which included 2014 Lee criteria (45 studies), the CTCAE criteria (15 studies), Penn criteria (8 studies), MSKCC criteria (2 studies), ASTCT (1 study), and unknown criteria used in the remaining 17 studies. However, there are some critical differences between these grading scales. For example, CTCAE were not adequately designed for evaluating CRS onset and severity while the ASTCT criteria address both CAR-T-cell-related CRS and NT. Assessment by the different grading systems will result in discordance in the incidences and severity of CAR-T therapy-associated CRS and NS [90].

This meta-analysis has a few limitations. First, the sample size of the studies we included varied greatly. A total of 19 out of 84 studies included less than 10 individuals, which resulted in small effect values and broad CIs. Second, we only performed subgroup analysis on factors that could be unified, such as age, region, and production process of CAR-T cells, to explore their potential impact on AEs. The tumor burden or efficacy that might be valuable but cannot be unified was not analyzed. In addition, the publication bias mainly focused on index text and patient selection. Since our study included various tumors, it was difficult to consider the identified reference standard as the best reference standard

## 5. Conclusions

The growing clinical trial and application of CAR-T therapy highlight the importance of the recognition and management of its unique toxicity profile. This study used meta-analysis to confirm that the incidences of common CAR-T related AEs such as CRS and NS were higher in patients with hematologic malignancies than in patients with solid malignancies, especially in patients with ALL and NHL. The major cause of CAR-T-related death was severe CRS due to early drug intervention, and the mortality rate was less than 1%. Common non-hematological toxicity including electrolyte imbalance, hyperpathia, capillary leak syndrome, coagulation dysfunction, and common NS manifestations including encephalopathy, dysphasia, and seizure were worthy of concern. Tumor type, CAR-T target, and dose infusion affect the occurrence of AEs. Patients’ age, race, and factors associated with production process of CAR-T such as vector were also potentially related to the occurrence of AEs. This global overview of CAR-T-related AEs can be used by clinicians as a reference that could guide clinical practice.

## Figures and Tables

**Table 1:** Characteristics of the included studies and participants.

First Author	Year	Cancer Type(s)	Patients Evaluated *n* (% Male)	Age, Years Mean (Range)	Country	CAR-T Type
Hematologic Malignancies						
Brentjens [21]	2011	CLL/ALL	8 (80)	63.9 (51–73)	USA	CD19
Brentjens [22]	2013	ALL	5 (80)	52.4 (23–66)	USA	CD19
Kochenderfer [23]	2013	CLL/DLBCL/MCL	10 (80)	52.4 (44–66)	USA	CD19
Dai [24]	2015	ALL	9 (44)	38.9 (15–65)	China	CD19
Brudno [25]	2016	CLL/DLBCL/MCL/ALL	20 (55)	48 (20–68)	USA	CD19
Locke [5]	2016	NHL	7 (NR)	NR	USA	CD19
Chen [26]	2017	ALL	6 (17)	26.5 (8–44)	China	CD19
Fitzgerald [27] *	2017	ALL	39 (51)	11 (5–22)	USA	CD19
Gardner [28]	2017	ALL	45 (51)	12.2 (1.3–25.3)	USA	CD19
Hay [18]	2017	ALL/CLL/NHL	133 (70)	54 (20–73)	USA	CD19
Kochenderfer [29] **	2017	DLBCL/MCL/FL	22 (NR)	47.4 (38–64)	USA	CD19
Neelapu [30] **	2017	NHL	110 (64)	58 (23–76)	USA	CD19
Pan [31]	2017	ALL	51 (63)	NR (2–68)	China	CD19
Schuster [32] *	2017	DLBCL/FL	28 (61)	NR (25–77)	USA	CD19
Lee [3]	2015	ALL/NHL	21 (66)	14.7 (5–27)	USA	CD19
Wei [33]	2018	ALL	23 (43)	35.8 (8–57)	China	CD19
Kochenderfer [4]	2015	NHL/CLL	15 (53)	56 (30–68)	USA	CD19
Park [34]	2018	ALL	53 (NR)	44 (23–74)	USA	CD19
Jin [35]	2019	ALL	8 (NR)	NR (30–81)	China	CD19
Ruark [36]	2019	ALL/NHL/CLL	40 (63)	54 (22–74)	USA	CD19
Ma [37]	2019	ALL	19 (40)	NR (3–13)	China	CD19
Yan [38]	2019	NHL	10 (80)	47 (32–59)	China	CD19
Nastoupil [39] **	2018	NHL	274 (NR)	60 (21–82)	NR	CD19
Jacobson [40] **	2018	NHL	104 (NR)	64 (21–84)	NR	CD19
Sano [41] **	2018	NHL	52 (NR)	42 (23–64)	NR	CD19
Spiegel [42] **	2018	DLBCL	22 (NR)	NR	NR	CD19
Jiang [43]	2019	ALL	58 (53)	NR	China	CD19
Bao [44]	2019	DLBCL	5 (NR)	NR (31–67)	China	CD19
Hu [45]	2018	ALL	31 (64)	31.6 (8–57)	China	CD19
Turtle [46]	2017	CLL	24 (NR)	61 (40–73)	USA	CD19
Maude [47] *	2018	ALL	75 (NR)	11 (3–23)	USA	CD19
Jacoby [48]	2018	ALL	20 (60)	11 (5–48)	Israel	CD19
Santomasso [19]	2018	ALL	53 (75)	NR	USA	CD19
Hirayama [49]	2019	FL	21 (67)	56 (51–62)	USA	CD19
Geyer [50]	2019	CLL/NHL	20 (70)	63 (43–75)	USA	CD19
Enblad [51]	2018	NHL/ALL	15 (46)	61 (24–71)	Sweden	CD19
Wang [52]	2019	ALL	5 (40)	31 (14–54)	China	CD19
Wang [53]	2016	DLBCL/MCL	16 (56)	60 (23–75)	USA	CD19
Turtle [1]	2016	ALL	30 (NR)	40 (20–73)	USA	CD19
Turtle [2]	2016	NHL	32 (84)	57 (22–70)	USA	CD19
Weng [54]	2018	ALL	3 (66)	20 (15–34)	China	CD19
Yan [55]	2019	MM	21 (48)	58 (49.5–61)	China	CD19 + BCMA
Hay [56]	2019	ALL	53 (57)	39 (20–76)	USA	CD19
Garfall [57]	2018	MM	12 (33)	61 (48–68)	USA	CD19
Ying [58]	2019	FL/DLBCL	25 (52)	NR (24–76)	China	CD19-BBz (86)
Cao [59]	2019	NHL	11 (NR)	65 (26–75)	China	CD19
Rossi [60]	2018	HL/NHL	22 (77)	NR (28–67)	USA	CD19
Rossig [61]	2017	ALL	11 (83)	9 (2–12)	Germany	CD19
Svoboda [62]	2018	HL	4 (NR)	NR (21–42)	USA	CD19
Kebriaei [63]	2016	NHL/ALL	26 (NR)	40 (21–61)	USA	CD19
Frey [64]	2019	ALL	35 (69)	34 (21–70)	USA	CD19
Hiramatsu [65] *	2019	ALL	6 (66)	(5–24)	Japan	CD19
Brudno [66]	2019	NHL	20 (NR)	NR	USA	CD19 (Hu19-CD828Z)
Wang [67]	2020	ALL/NHL	89	36 (9–71)	China	CD19/22
Abramson [68] ***	2020	DLBCL	269 (65)	63 (54–70)	USA	CD19
Liu [69]	2020	CLL/NHL	11 (63)	60 (47–70)	USA	CD19 CAR–NK
Till [70]	2008	idon–NHL	9 (89)	60.2 (43–77)	USA	CD20
Till [71]	2011	idon-NHL	4 (100)	59.2 (28–80)	USA	CD20
Wang [72]	2014	DLBCL	7 (85)	62.4 (37–85)	China	CD20
Curran [73]	2019	ALL	25 (NR)	(1–22.5)	USA	CD20
Fry [6]	2017	ALL	21 (62)	19 (7–30)	USA	CD22
Shalabi [7]	2018	ALL/DLBCL	22 (63)	17.9 (7.3–30.5)	USA	CD22
Ramos [74]	2017	HL	9 (67)	34.6 (20–65)	USA	CD30
Wang [75]	2017	HL	18 (72)	33 (13–77)	China	CD33
Ritchie [76]	2013	AML	5 (33)	70.6 (64–78)	Australia	LeY
Zhao [8]	2018	MM	57 (60)	54 (27–72)	China	LCAR-B38M
Ali [9]	2016	MM	12 (NR)	NR	USA	BCMA
Brudno [10]	2018	MM	16 (NR)	NR	USA	BCMA
Raje [11]	2019	MM	33 (64)	60 (37–75)	USA	BCMA
Cohen [12]	2019	MM	25 (68)	58 (44–75)	USA	BCMA
Baumeister [77]	2018	AML/MDS/MM	12 (75)	70 (44–79)	USA	NKG2D
Solid Malignancies						
Katz [78]	2015	Liver Cancer	6 (67)	57 (51–66)	USA	CEA
Zhang [79]	2017	Colorectal Cancer	10 (70)	58 (48.8–67)	China	CEA
Feng [15]	2016	Non Small Cell Lung Cancer	11 (45)	58 (40–66)	China	EGFR
Guo [80]	2017	Biliary Tract Cancer	19 (53)	57 (39–70)	China	EGFR
Goff [81]	2019	Glioblastoma	18 (83)	NR (43–64)	USA	EGFRvIII
Louis [16]	2011	Neuroblastoma	19 (47)	7 (3–20)	USA	GD2
Ahmed [82]	2015	Sarcoma	19 (47)	17 (7.7–29.6)	USA	HER2
Feng [17]	2017	Biliary Tract Cancer /Pancreatic Cancer	11 (82)	60.5 (50–75)	China	HER2
Brown [13]	2015	Glioblastoma	3 (NR)	NR	USA	IL13-zetakine
O’Rourke [14]	2017	Glioblastoma	10 (50)	59.5 (45–76)	USA	Mesothelin
Haas [83]	2019	Pleural Mesothelioma /Ovarian Carcinoma /Pancreatic Cancer	15 (67)	69 (48–75)	USA	Mesothelin
Junghans [84]	2016	Prostate Cancer	5 (100)	61 (51–75)	USA	PSMA
Hege [85]	2017	Colorectal Cancer	14 (NR)	NR	USA	TAG-72

* The CAR-T is tisagenlecleucel (CTL-019, Kymriah) from Novartis Pharma (approved by FDA); ** The CAR-T is axicabta geneciloleucel (KTE-C19, Yescarta) from Kite Pharma (approved by FDA); *** The CAR-T is lisocabtagene maraleucel (a novel CD19-directed CAR T-cell with a 4-1BB co-stimulatory domain administered as sequential infusions of equal target doses of CD8+ and CD4+ CAR+ T cells) from Bristol–Myers Squibb. FL, follicular lymphoma; DLBCL, diffuse large B lymphoma; NHL, non-Hodgkin’s lymphoma; HL, Hodgkin’s lymphoma; ALL, acute lymphoblastic leukemia; MM, multiple myeloma; CLL, chronic lymphocytic leukemia; idon-NHL, indolent non-Hodgkin’s lymphoma; and MDS, myelodysplastic syndrome.

## Data Availability

Not applicable.

## Abbreviations

CAR-T: Chimeric antigen receptors T cells; CRS: cytokine release syndrome; NS: neurological symptoms; MM: multiple myeloma; ALL: acute lymphoblastic leukemia; CRES: CAR-T-cell-related encephalopathy syndrome; AEs: adverse events; ORs: odds ratios; BCMA: B-cell maturation antigen; NKG2D: natural killer group 2D; CEA: carcinoembryonic antigen; EGFR: epidermal growth factor receptor; HER2: epidermal growth factor receptor 2; PSMA: prostate specific membrane antigen; TAG72: tumor-associated glycoprotein-72; TF: tissue factor; PECAM-1: platelet endothelial cell adhesion molecule-1.

## Appendix A

**Table A1:** Causes of 53 treatment-related deaths in clinical trials of CAR-T.

**Cause of Death**	**No. (%)**
Cytokine release syndrome	23 (43.4)
Neurological symptoms	8 (15.1)
**Respiratory**	
pneumonia	3 (5.6)
Pulmonary embolism	2 (3.7)
**Cardiovascular**	
Cardiac arrhythmia	1 (1.9)
Heart failure	2 (3.7)
**Infectious**	
Sepsis	5 (9.4)
**Coagulation dysfunction**	
Hemorrhage	8 (15.1)
aGVHD	1 (1.9)
